# Sex-specific associations between body mass index and death before life expectancy: a comparative study from the USA and Sweden

**DOI:** 10.1080/16549716.2019.1580973

**Published:** 2019-04-05

**Authors:** Melissa Scribani, Margareta Norberg, Kristina Lindvall, Lars Weinehall, Julie Sorensen, Paul Jenkins

**Affiliations:** a Bassett Healthcare Network, Bassett Research Institute, Cooperstown, NY, USA; b Epidemiology and Global Health, Department of Public Health and Clinical Medicine, Umeå University, Umeå, Sweden

**Keywords:** Obesity, premature mortality, longitudinal studies, all-cause mortality, circulatory disease mortality

## Abstract

**Background**: Understanding the impact of obesity on premature mortality is critical, as obesity has become a global health issue.

**Objective**: To contrast the relationship between body mass index (BMI) and premature death (all-cause; circulatory causes) in New York State (USA) and Northern Sweden.

**Methods**: Baseline data were obtained between 1989 and 1999 via questionnaires (USA) and health exams (Sweden), with mortality data from health departments, public sources (USA) and the Swedish Death Register. Premature death was death before life expectancy based on sex and year of birth. Within country and sex, time to premature death was compared across BMI groups (18.5–24.9 kg/m^2^ (reference), 25–29.9 kg/m^2^, 30.0–34.9 kg/m^2^, ≥35.0 kg/m^2^) using Proportional Hazards regression. Absolute risk (deaths/100,000 person-years) was compared for the same stratifications among nonsmokers.

**Results**: 60,600 Swedish (47.8% male) and 31,198 US subjects (47.7% male) were included. Swedish males with BMI≥30 had increased hazards (HR) of all-cause premature death relative to BMI 18.5–24.9 (BMI 30–34.9, HR = 1.71 (95% CI: 1.44, 2.02); BMI≥35, HR = 2.89 (2.16, 3.88)). BMI≥25 had increased hazards of premature circulatory death (BMI 25–29.9, HR = 1.66 (1.32, 2.08); BMI 30–34.9, HR = 3.02 (2.26, 4.03); BMI≥35, HR = 4.91 (3.05, 7.90)). Among US males, only BMI≥35 had increased hazards of all-cause death (HR = 1.63 (1.25, 2.14)), while BMI 30–34.9 (HR = 1.83 (1.20, 2.79)) and BMI≥35 (HR = 3.18 (1.96, 5.15)) had increased hazards for circulatory death. Swedish females showed elevated hazards with BMI≥30 for all-cause (BMI 30–34.9, HR = 1.42 (1.18, 1.71) and BMI≥35, HR = 1.61 (1.21, 2.15) and with BMI≥35 (HR = 3.11 (1.72, 5.63)) for circulatory death. For US women, increased hazards were observed among BMI≥35 (HR = 2.10 (1.60, 2.76) for all-cause and circulatory HR = 3.04 (1.75, 5.30)). Swedish males with BMI≥35 had the highest absolute risk of premature death (762/100,000 person-years).

**Conclusions**: This study demonstrates a markedly increased risk of premature death associated with increasing BMI among Swedish males, a pattern not duplicated among females.

## Background

Obesity is a significant global health issue affecting high, middle, and low-income nations alike [1–3]. Among US adults, the prevalence of those affected by obesity (body mass index (BMI) of ≥30.0 kg/m^2^) has risen from 14.5% in the early 1970s [4] to 35.7% in 2009–2010 [5], and to 36.5% in 2014 [6]. In Sweden, the prevalence of adults affected by obesity is progressing at a more modest pace; the prevalence was 15% in 2016, up from 11% in 2004 [7]. Several low- and middle-income countries had surpassed even the US prevalence as of 2013, with reports as high as 50% of adults affected by obesity [8].

The severe adverse impacts of obesity on overall health and quality of life have been well documented in several countries [9–17]. Significant associations between obesity and increased mortality from all causes [18–21], and from cardiovascular disease, stroke, and malignancy [18,21,22] have also been reported. Evidence is conflicting regarding the association between overweight (BMI 25.0–29.9 kg/m^2^) and mortality [19], although several studies document increased hazards of all-cause mortality associated with any level of BMI exceeding 18.5–24.9 kg/m^2^ [18,23–25].

While previously published analyses include data from global regions [18,25], very few directly compare country-by-country, sex-specific patterns of the relationship between BMI and premature mortality. Published studies have noted sex differences in BMI obesity prevalence and comorbidities associated with obesity [18,26,27]; therefore, sex-specific analyses are important when considering associations with mortality. The impact of obesity on premature death is particularly relevant from the standpoint of prevention and intervention planning. Various definitions of premature death have been published [28], including expressing premature death as years of potential life lost (YPLL) or death before a given cut-off age [24]. Several studies have shown obesity to be associated with significantly increased all-cause mortality before age 70 [24,29].

Considering the significant impacts of elevated BMI on health, and the rapid global development of obesity in high-, middle-, and more recently, low-income countries, study of the relationship between obesity and premature death in areas with varying degrees of obesity prevalence [8,30,31] is relevant to the understanding of the potential consequences of obesity worldwide. In the USA, more than one in three adults is affected by obesity; in Sweden, fewer than one in seven adults is affected. This study provides an opportunity to contrast the patterns of BMI-related premature death in two locations that have differential distributions of obesity, but have similar resources. Studying the relationship between obesity and premature death across these two settings is an important contribution to global health, as this work may stimulate further comparisons of this type between additional countries and regions, and further exploration into the drivers of potential differences or how the impacts of obesity differ in various contexts.

We present an analysis of robustly defined premature death (death before life expectancy), from all causes and from circulatory diseases, to quantify the undue burden of obesity in two regions that are economically similar but have clear contextual differences in terms of BMI distribution and obesity prevalence. We address the sex-specific patterns of association between BMI and the risk of premature death observed in US and Swedish populations. This study is the first to elucidate cross-country, sex-specific patterns of risk of premature death defined in this manner.

## Methods

### Study design and settings

This is a longitudinal comparison of two adult cohorts, one from a mainly rural seven-county region of central New York (USA), and the other from Västerbotten County, Sweden, a largely rural region with two urban areas (Umeå and Skellefteå).

The US cohort included inhabitants of Chenango, Delaware, Herkimer, Madison, Montgomery, Otsego and Schoharie Counties. Based on urban/rural designations defined by the 2010 US Census, the study counties are more than 90% rural. There are 14 urban clusters (population 2500–50,000) within the study counties, with only two municipalities of greater than 10,000 inhabitants [32,33]. The included counties are served by an integrated health care system that includes an academic medical center, four affiliate hospitals, skilled nursing facilities, and more than two dozen primary care clinics distributed throughout the region. This region has been noted to have high prevalence of cardiovascular disease and relevant risk factors, including obesity [34].

Swedish subjects were residents of Västerbotten County, home to the Västerbotten Intervention Program (VIP), an integrated community health program focused on lowering cardiovascular disease, which has been shown to be higher in this county than other counties in Sweden [35,36]. Västerbotten is comprised of many small towns and villages, as well as the one urban area (defined consistently with the US Census) of Umeå (population 112,000 in 2007) and the largest urban cluster of Skellefteå (population 38,000). Västerbotten is home to 32 primary health care facilities, two regional hospitals and one University hospital.

These two settings present an opportunity for comparison of high-quality data sets from resource-rich environments with documented elevated risk for cardiovascular disease. Existing contextual differences, specifically, differing BMI trends and obesity prevalence, provide an interesting contrast.

### US subjects

Adults participating in the 1989 Bassett Health Census (BHC) or the 1999 BHC, aged 28–65 at the time of the survey, were included [34,37]. This age range was chosen to align with the corresponding ages of Swedish subjects. With a response rate of 84%, 17,147 households participated in the 1989 BHC [37]. The 1999 BHC had a response rate of 75.1%, with 24,106 households responding. All data for the BHC studies were self-reported; height and weight were adjusted for self-report bias using methodology described by Scribani et al. [38].

Briefly, using measured height and weight paired with self-reported data, sex-specific linear regression equations were developed to predict measured values. The coefficients from these equations were then applied to self-reported data to minimize reporting bias.

### Swedish subjects

Individuals participating in the Västerbotten Intervention Program (VIP) between 1989 and 1999 were included. The VIP was introduced in 1985 in Norsjö municipality and was expanded to all of Västerbotten county by 1992 [35,36]. Subjects were invited to participate in the program at age 30 (until 1995), 40, 50, and 60 as part of regular primary care. Height and weight were measured during a physical examination, and demographic data were gathered by questionnaire. Participation rates range from 48% to 69% [36], and approximately 167,000 health examinations had been performed as of 2015.

### Endpoints

The outcomes were premature death from any cause and from circulatory causes (underlying cause of death ICD9: 390–459 and ICD10: 100-199). A premature death occurred if the subject died prior to reaching the life expectancy based on their year of birth and sex. For US subjects, these life expectancies were taken from the Centers for Disease Control life expectancy tables [39]. The life expectancy of Swedish subjects was obtained from the Swedish Central Bureau of Statistics [40]. Death prior to life expectancy allows for sensitivity to the changing life expectancies among a cohort that was enrolled at various ages and followed for several decades. Life expectancies ranged from 53.6 to 66.8 years among US males, from 54.6 to 74.3 years among US females, from 55.6 to 71.7 years among Swedish males and from 58.4 to 76.1 years among Swedish females.

In Sweden, date of death and underlying cause were obtained from the Swedish National Board of Health and Welfare’s cause of death register. This death register has been shown to be virtually 100% complete and of very high quality [41]. In the US, death records were provided by the New York State Department of Health (NYS DOH) and the New York City Department of Health and Mental Hygiene. To validate the completeness of the US data, any subject whom the state indicated was not deceased and was at least 40 years of age as of June 2007 was included in a secondary data search. This process involved consulting with the Social Security Death Index, Ancestry.com, local obituaries, and newspaper clippings. This investigative process identified 24.1% of the total deaths.

For those US subjects who died prematurely, person-years of follow-up were calculated from the date of earliest study participation until the date of death. The right censoring date for US subjects was the later of either (a) the date of receipt of the files from NYS DOH, or (b) the latest date of the manual investigation (concluded in 2011). Subjects who reached life expectancy prior to either (a) or (b) were right censored as of the date of reaching life expectancy.

In Sweden, for subjects who died prematurely, follow-up time was calculated from the date of earliest participation in VIP to the date of death. All other subjects were right censored at the earlier of the date of death registry file (May 2016) or the date of reaching life expectancy.

All covariate data were taken from the BHC or VIP accordingly. BMI strata were: BMI<18.5 kg/m^2^, 18.5–24.9 kg/m^2^, 25.0–29.9 kg/m^2^, 30.0–34.9 kg/m^2^, and BMI≥35.0 kg/m^2^. Age (continuous), smoking status (any current smoking versus nonsmoker), and education (university versus less than university) were also included. Individuals with BMI<18.5 kg/m^2^ were ultimately excluded from analyses due to limited numbers of subjects.

The appropriate ethical review boards approved each study. The Swedish study was approved by the Umeå University ethical review board (approval numbers Dnr 08-113M and 2014-196-32M), and the US study was approved by the Mary Imogene Bassett Hospital Institutional Review Board (approval numbers 491 and 925). For the Swedish study, consent was implied via participation in the VIP. Participants were informed that exam data would be linked to mortality information, and were offered the opportunity to withdraw. For the US study, consent was implied via return of the questionnaire.

### Analytic models

Proportional Hazards regression (PH) was used to analyze time to all-cause premature death and circulatory-caused premature death using three model structures (noted as Models 1, 2, and 3). The assumption of proportional hazards was tested by including covariate by time interaction terms in the models. In cases where these terms were significant, a plot of the survival curves across each level of the covariate was inspected for disordinal interaction. There were no meaningful departures away from the proportional hazards assumption, and retention of the covariate by time interaction terms was unnecessary.

Because published studies have documented differences in mean BMI and obesity prevalence, as well as obesity-related comorbidities between men and women [26,27], all analyses were stratified by sex with the exception of Model 3, described below. Age at entry into the study, education, and smoking were included as covariates in all models. Due to differences in measurement methodology between countries, additional potentially confounding variables, such as physical activity, comorbid conditions, overall health status, alcohol use, and other behaviors were not included.

To assess the dose response relationship between BMI strata and each endpoint (with BMI 18.5–24.9 kg/m^2^ as the reference stratum), the initial PH model (Model 1) was stratified by sex and country. To obtain a formal statistical test of interaction, i.e. a differential change in hazard ratios across BMI strata between the two countries, a multiplicative interaction term of country by BMI stratum was created (Model 2). In this analysis, data from both countries were combined, but were stratified by sex. Finally, a model that included all subjects was specified that included a three-way interaction of country by sex by BMI stratum (Model 3). This was used to obtain a formal statistical test of whether the change across BMI strata between countries differed by sex.

### Absolute risk of premature death

Premature mortality risk across BMI strata was estimated using the incidence density (number of premature deaths per 100,000 person-years) of premature death separately by country and sex. These analyses were limited to nonsmokers, due to the disproportionately high prevalence of smoking among US males.

### Proportional hazards regression sensitivity analyses

A sensitivity analysis using death before age 70 as the outcome was conducted as a means of comparing our results to studies using this alternate definition of premature death. In addition, we constructed PH models stratified by smoker/nonsmoker. Also, the fact that subjects in the US study enrolled during two cross-sectional periods (1989 or 1999) may pose a limitation, if the pattern of the association between obesity and premature death had somehow changed during those 10 years. To assess this, we split subjects into early (1989–1994) versus late (1995–1999) cohorts.

## Results

A total of 31,198 subjects were included from the USA, contributing a combined 409,804 years of follow-up (mean follow-up = 13.1 years); 60,600 subjects from Sweden were included, contributing a total of 943,555 years of follow-up (mean follow-up = 15.6 years). Demographics for each country’s cohort are displayed in Table 1. As shown, the proportion of females was slightly greater than 50% in both Sweden and the USA, with virtually identical mean ages in the two countries. Smoking prevalence was markedly higher in the USA (28.7% versus 17.6% in Sweden), and the proportion of subjects receiving university education was slightly lower in Sweden. The overall mean BMI in the Swedish cohort was 1.7 kg/m^2^ less than for the US cohort at 25.6 and 27.3 kg/m^2^, respectively.

**Table 1.** Demographic Characteristics of Subjects, Sweden and USA.

	Sweden	USA
Total number of subjects	60,600	31,198
**Males**	28,947 (47.8%)	14,884 (47.7%)
**Females**	31,653 (52.2%)	16,314 (52.3%)
**Age at enrollment, mean (SD)**	46.6 (9.9)	44.4 (9.0)
**Education**		
Less than University	46,211 (77.4%)	23,020 (74.3%)
University	13,513 (22.6%)	7,975 (25.7%)
**Current smoking**		
Nonsmoker	49,958 (82.4%)	21,703 (71.4%)
Smoker	10,642 (17.6%)	8,715 (28.7%)
**Body mass index, kg/m^2,^ mean (SD)**	25.6 (9.9)	27.3 (5.5)
**BMI stratum**		
18.5–24.9	30,181 (49.8%)	11,997 (38.5%)
25.0–29.9	23,347 (38.5%)	11,533 (37.0%)
30.0–34.9	5,635 (9.3%)	5,096 (16.3%)
35.0+	1,437 (2.4%)	2,572 (8.2%)

For both males and females, a significantly larger proportion of subjects had BMI≥30 in the USA compared to Sweden (Table 2). In Swedish males, only 1.5% of study subjects had BMI≥35 versus 7.7% of US male subjects. Among both males and females, the average BMI within strata was markedly similar between the US and Sweden. The largest sex-specific difference in mean BMI was among females with BMI≥35, where the mean BMI in the USA was roughly 2 kg/m^2^ greater than Sweden. Crude death rates (all-cause premature death) among Swedish men increased from 4.2% for subjects with BMI 18.5–24.9 to 7.3% among those with BMI 30–34.9 and 11.5% among those with BMI≥35. In US men, a higher crude death rate among BMI 18.5–24.9 (5.3%) attenuated among BMI 30–34.9 (4.8%), and rose to 7.1% among those with BMI≥35. Men’s premature circulatory crude death rates showed a smaller magnitude, but overall similar pattern of association as BMI increased. Crude death rates for both all-cause premature death and premature circulatory death among women were markedly similar between the Sweden and the USA.

**Table 2.** Distribution of BMI Strata by Country and Sex, Mean BMI (kg/m^2^) Within Each Stratum, and Number of Premature Deaths (Due to All Causes and Circulatory Causes).

	*Sweden*				*USA*			
	N (%)	Mean BMI	All-cause deaths, N (%)	Circulatory deaths, N (%)	N (%)	Mean BMI	All-cause deaths, N (%)	Circulatory deaths, N (%)
MALES BMI (kg/m^2^)								
18.5–24.9	12,445 (43.0)	23.0	525 (4.2)	121 (1.0)	3,627 (24.4)	23.3	192 (5.3)	38 (1.0)
25.0–29.9	13,444 (46.4)	27.0	669 (5.0)	235 (1.7)	7,102 (47.7)	27.3	313 (4.4)	102 (1.4)
30.0–34.9	2,613 (9.0)	31.7	191 (7.3)	81 (3.1)	3,017 (20.3)	32.0	145 (4.8)	55 (1.8)
35.0+	445 (1.5)	38.7	51 (11.5)	20 (4.5)	1,138 (7.7)	39.5	81 (7.1)	34 (3.0)
FEMALES BMI (kg/m^2^)								
18.5–24.9	17,736 (56.0)	22.4	603 (3.4)	78 (0.4)	8,370 (51.3)	22.1	213 (2.5)	37 (0.4)
25.0–29.9	9,903 (31.3)	27.0	373 (3.8)	56 (0.6)	4,431 (27.2)	27.2	160 (3.6)	27 (0.6)
30.0–34.9	3,022 (9.6)	31.9	149 (4.9)	25 (0.8)	2,079 (12.7)	32.2	80 (3.8)	24 (1.2)
35.0+	992 (3.1)	38.4	53 (5.3)	14 (1.4)	1,434 (8.8)	40.4	75 (5.2)	21 (1.5)

For US males, there was a 2.5-fold greater prevalence of smokers (38.6% smokers) among those with BMI 18.5–24.9 as compared to the same BMI stratum in Swedish men (15.7% smokers). Among females, a higher proportion of those in the USA were smokers as compared to Sweden, across all BMI strata. This disproportionality prompted sensitivity analyses (proportional hazards modeling) including only nonsmokers.

### Relative hazard of premature death

**Figure 1.**
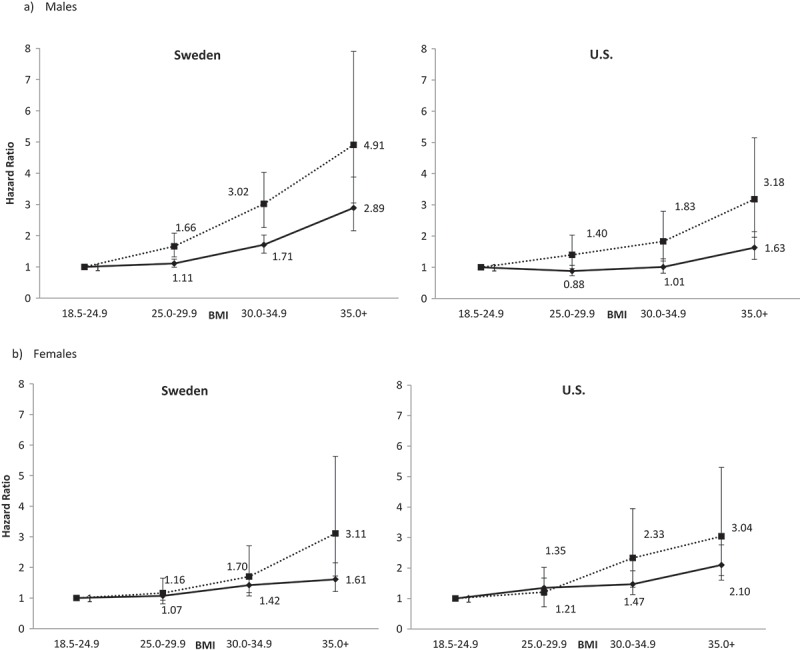
Hazard ratio gradient across BMI strata for (a) males and (b) females. Solid lines indicate hazard ratios for all-cause premature death; dotted lines indicate hazard ratios for premature circulatory death. Shaded labels indicate p < 0.05 relative to BMI = 18.5–24.9.

For males in general, there was a steeper gradient in the rise of the hazard of premature death as a function of BMI in Sweden than in the USA (Figure 1(a)). For all cause premature death in US males, relative to BMI 18.5–24.9, the hazard ratios for BMI 25.0–29.9 (HR = 0.88, 95% Confidence Interval (CI) 0.73, 1.06) and BMI 30.0–34.9 (HR = 1.01, 95% CI 0.81, 1.27) were not statistically significant; however, the hazard ratio was significant for BMI≥35 (HR = 1.63, 95% CI 1.25, 2.14). The slope of this rise was much more moderate than the corresponding increase in Sweden, where the hazard ratios rose monotonically from HR = 1.11 (95% CI 0.99, 1.25) among BMI 25.0–29.9 to HR = 2.89 (95% CI 2.16, 3.88) among BMI≥35. The pattern of hazard ratios progressing from BMI 25.0–29.9 to BMI≥35 were markedly more divergent between the USA and Sweden for premature circulatory death.

For females (Figure 1(b)), the pattern of hazard ratios for all-cause premature death across BMI strata relative to BMI 18.5–24.9 was very similar between the USA and Sweden, with a slight increase to HR = 2.10 (95% CI 1.60, 2.76) in the USA for BMI≥35. For premature circulatory death, the slope of the change from BMI 25.0–29.9 to BMI 30.0–34.9 was steeper than that observed in Sweden, rising to HR = 2.33 (95% CI 1.37, 3.95) for BMI 30.0–34.9.

The interaction terms in the sex-specific models that combined data from the two countries were significant for males (all-cause, p = 0.002, circulatory, p = 0.03). Although the divergence in the pattern of the hazard ratios between the two countries is greater for circulatory death, the significance levels actually become weaker due to smaller numbers of events (see Table 2). These interaction terms were not significant for females for all cause death (p = 0.97) or circulatory death (p = 0.63). There was a significant three-way interaction of sex by country by BMI stratum for premature circulatory death (p = 0.008), as demonstrated by Figure 1(a,b).

### Absolute risk of premature death

**Figure 2.**
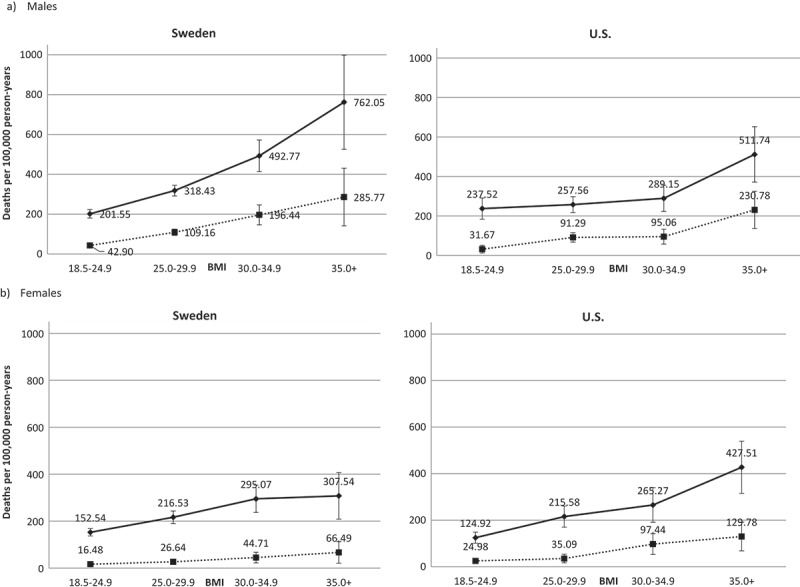
Incidence density of premature death (per 100,000 person-years) across BMI strata for (a) male nonsmokers and (b) female nonsmokers. Solid lines depict all-cause premature death, and dotted lines depict premature circulatory death.

Among nonsmoking males, the absolute risk of premature death by any cause was lower in Sweden (202/100,000 person-years) than in the USA (238/100,000 person-years) for those with BMI 18.5–24.9 (Figure 2(a)). With increasing BMI, however, the absolute risk of premature death surpassed those in the respective US groups. Among those with BMI≥35, the risk of premature death in Sweden was 762/100,000 person-years versus 512/100,000 person-years in the USA.

Among females, the absolute risk of premature death from all causes and circulatory causes in subjects with BMI 18.5–24.9 was very similar between the countries (Figure 2(b)). The rise in absolute risk of premature death from all causes, and, to a lesser degree, premature death from circulatory causes was more pronounced among US women than Swedish women when progressing from BMI 18.5–24.9 to BMI≥35.

### Sensitivity analyses

Using death before age 70 as an alternate outcome definition, the pattern of hazard ratios was very similar to the main analysis, although the trajectory of hazards associated with increasing BMI was not as steep. Among men, the hazard of premature all-cause death was significantly elevated for each classification of BMI≥30 in Sweden relative to BMI 18.5–24.9 (HR = 1.56 and 2.17 for BMI 30–34.9 and BMI≥35, respectively). Among US men, only modestly increased hazards of premature all-cause death were observed, and only the hazard ratio among BMI≥35 (HR = 1.27) reached statistical significance. For circulatory death before age 70, there was a statistically significant interaction for the hazard ratio pattern between Sweden and the USA. The hazard ratio rose to 3.51 among men with BMI≥35 relative to BMI 18.5–24.9 in Sweden, whereas the corresponding hazard in US men was 2.15. For women, the hazards associated with increasing BMI for death before age 70 from all causes was nearly identical to those using death before life expectancy as the outcome in both Sweden and the USA. For circulatory death before age 70, the hazards were attenuated as compared to the death before life expectancy outcome (HRs for BMI≥35 relative to BMI 18.5–24.9 were 2.72 and 2.34 for Swedish and US women, respectively). As with the main analysis, there was no evidence of interaction effect of country by BMI group for women.

Proportional hazards models among nonsmokers demonstrated an identical pattern to what was seen in the full models, albeit with slightly steeper trajectories for the association in both the USA and Sweden. The country by BMI group interaction terms among male nonsmokers were p = 0.002 and p = 0.065 for all-cause and circulatory premature mortality, respectively. Proportional hazards models among nonsmoking females produced very similar results to the main analyses. The results of the early/late enrollment cohort analysis produced comparable findings to the full analysis, and therefore, no detailed results are presented.

## Discussion

This analysis demonstrates that Swedish males appear to be at the greatest increased risk for premature death (both overall and from circulatory-related causes) as BMI increases relative to BMI 18.5–24.9. They also experienced the steepest absolute risk gradient as contrasted with US males or females from either country.

The crude survival advantage among Swedish nonsmoking males with BMI 18.5–24.9 over US males with BMI 18.5–24.9 disappeared at BM≥25 kg/m^2^ for all-cause premature death. Adjusted for age, smoking, and education, hazard ratios for the association between premature death and BMI≥30 kg/m^2^ relative to BMI 18.5–24.9 kg/m^2^ were strongest among Swedish males for all causes and circulatory causes.

Increasing BMI was associated with significantly increased hazards of premature death among females in the USA and Sweden; however, the model combining countries revealed no significantly different pattern. Among nonsmoking females, the crude survival advantage among BMI 18.5–24.9 kg/m^2^ Swedes is minimal for premature circulatory deaths, although Swedish women with BMI≥35 kg/m^2^ had a lower absolute risk of all-cause premature death than US females in that same BMI stratum.

The mean BMI within BMI strata did not differ significantly between the USA and Sweden for either males or females. Therefore, the disparities cannot be explained by a disproportionately ‘heavier’ group of BMI≥35 kg/m^2^ men in Sweden than the USA, or, conversely, by a disproportionately ‘lighter’ group of Swedes in the BMI 18.5–24.9 kg/m^2^ stratum.

A greater prevalence of smoking, particularly among US males compared to Swedish males is one potential explanation for the differential hazard ratio patterns seen with increasing BMI relative to normal weight within each country. On sensitivity analysis, PH models among nonsmokers showed a similar pattern to the full models, but with steeper trajectories for the association in both the USA and Sweden. Therefore, the high prevalence of smoking in US men, most notably among BMI 18.5–24.9 kg/m^2^, does not account for the dramatically different patterns in hazard ratios between the two countries.

While this observational study cannot attribute causal influence as to why the trajectory of hazards for BMI≥30 kg/m^2^ relative to BMI 18.5–24.9 kg/m^2^ is so much more pronounced for Swedish males, it may relate to physical activity, which we were unable to control for. US men with BMI≥30 kg/m^2^ have been shown to engage in more minutes of physical activity per day than Swedish men in the same BMI stratum [42]. Conversely, in that same study, Swedish women were physically active for more minutes per day than US women at each level of BMI. Women in Sweden may also have a higher level of health consciousness regardless of BMI. A recent study from Sweden demonstrated that healthy behaviors and healthy eating were more common among Swedish women than US women, even for those who were affected by obesity [43].

There may be a myriad other possibilities with respect to differential overall health and well-being among males with BMI≥35 in Sweden versus the USA. In Sweden, severe obesity may be associated with adverse health outcomes due to stigmatization and other social inequities. It has been well-established that stigmatization is associated with obesity in US contexts [44], and Swedish studies have also shown increased healthcare and workplace stigmatization among those affected by severe obesity [45,46]. While our study used education as a proxy for socioeconomic status, we did not have data on other socioeconomic factors. Obesogenic surroundings [47–49] may play a role; however, this is difficult to explain, considering the strength of the relationship between obesity and premature death among females in our study did not mirror that among males.

We believe that our definition of premature death as any death prior to life expectancy is highly robust and a significant strength of our study, particularly considering that life expectancy had increased over the study period. While years of productive life lost (YPLL)-based studies take a complementary approach, we opted to focus on premature death as the outcome because this was not designed as a study about longevity. Our definition allows for an accurate representation of a truly premature death. In addition, while the follow-up period in Sweden extended for a few years beyond that in the USA, the use of death before life expectancy as the outcome allows us to take advantage of the full range of follow-up. Because this analysis is mainly a comparison of trends (i.e. how the increased risk of premature death changes within a country as a function of increasing BMI), the comparisons between BMI categories within each country are using a common definition of premature death that applies equally across BMI strata.

An additional strength is the data validation that was undertaken for US death information. Dates and causes of death from the NYS DOH were verified using a number of other sources to minimize reporting error in the US sample. Achieving completeness in death status (nearly one-quarter of the deaths in the US cohort were identified through this validation process) and cause of death was labor-intensive, but provided a level of sophistication in this data set that is rare among US mortality studies.

Direct comparison of these trends between males and females is complicated by the much greater number of premature deaths, and consequently greater statistical power, in the models for the males. This does not appear to be an issue for all-cause death, as the steeper gradient seen in Swedish males over their American counterparts is clearly not present for the females. However, for circulatory deaths (males 686, females 282), this may have produced some level of instability in the models.

Despite the technical challenges in comparing cross-country trends between males and females, it is clear that there are sex-related nuances in the relationship between premature death and increasing BMI that cannot be ignored. Sex-stratified analyses like those presented here are critical to understanding these associations. Recently published pooled studies include sex-specific modeling as well [18,25].

Our results among males corroborate studies demonstrating greater hazards of all-cause death associated with higher BMI in Europe as compared to North America [18]. Our sensitivity analysis restricted to nonsmokers produced similar patterns in the relationship of premature all-cause death to those shown in a recent large meta-analysis of overall mortality [18,25]. These same studies also concur with our finding of an attenuated rise in hazard of death for women affected by obesity and severe obesity relative to normal weight women as compared to men. The hazard ratios observed in our study associated with BMI≥25 for Sweden and the US men were of similar magnitude to those reported from European and North American regions for men. However, for women, we observed higher hazard ratios (on average 0.75 to 1.0 HR units greater in our study for BMI≥30 as compared to European and North American regions) [18].

Our findings may have significant implications for the focus of public health initiatives across the globe, particularly in nations where there is a more modest prevalence of those affected with obesity as compared to the USA. The apparent increased risk of premature death among Swedish men affected by severe obesity points to an urgent need for clinical interventions with this group. In terms of slowing the obesity epidemic in hopes of preventing future premature deaths, numerous wide-ranging programs aimed at weight loss among those affected by obesity have been largely unsuccessful [50]. Focused interventions promoting a healthy weight and primary prevention of obesity, or primary weight maintenance [51], may be the most promising tactic for lessening the future burden of premature death in high-, middle-, and low-income countries alike.

### Limitations

The methodology with which independent and dependent variables were collected was not identical between the countries. However, it is highly unlikely that this accounted for the differences seen in the relationship between premature death and BMI. Assuming that the Swedish death data are more complete [41], we would expect a higher overall death rate in Sweden as compared to the USA. Furthermore, identifying a death would not be related to the subject’s BMI differentially between countries.

In the US data, height and weight were self-reported; however, these values were corrected using a linear regression equation [38]. While measured height and weight would have been optimal, the correction for self-reported height and weight among US subjects helped to offset any bias introduced via self-report.

We acknowledge the weakness in associating only baseline BMI (and covariates) with premature death. In particular, BMI and smoking status may have changed over the course of follow-up. We were also unable to include controlling variables for comorbid conditions, such as baseline heart disease or hypertension, or behaviors, such as physical activity or alcohol use. These factors were not measured in a consistent manner between countries, and therefore could not be formally analyzed.

Additional selection biases cannot be discounted totally, considering there was not 100% participation in either the VIP health exams (Sweden) or the BHC questionnaires (USA). However, published reports have shown minimal social selection to be associated with either of these studies [35,52]. Further, it is unlikely that electing to participate in the studies would be related to the association between BMI and risk of premature death.

## Conclusions

The increased risk of premature all-cause and circulatory death associated with increasing BMI relative to normal weight among Swedish males was markedly greater than the corresponding increased risk for US males, a pattern not duplicated among females. Investigation into the factors underlying this phenomenon among males may provide further understanding of the mechanisms of obesity-related premature mortality. Primary prevention of obesity, that is, maintenance of a healthy BMI, should remain a focus of public health initiatives in both countries to mitigate the risk of premature death, and may be most impactful if prioritized in Swedish males.

## Paper context

Few studies have contrasted obesity-linked mortality across individual countries. We found clear vulnerabilities among severely obese men in Sweden, up to a fivefold increased risk of premature death versus normal weight. In US men, the risk was much more moderate. No such disparity was found among females. Research to explain the mechanism of these gender-specific differences between countries may inform prevention initiatives globally. This, in turn, would help to mitigate the obesity-related risk of premature death.
